# Awareness of heart disease and associated health behaviours in a developing country: A qualitative study

**DOI:** 10.1002/nop2.961

**Published:** 2021-06-12

**Authors:** Lemma N. Bulto, Judy Magarey, Philippa Rasmussen, Jeroen M. L. Hendriks

**Affiliations:** ^1^ College of Health and Medical Sciences Haramaya University Harar Ethiopia; ^2^ Adelaide Nursing School University of Adelaide Adelaide SA Australia; ^3^ Caring Futures Institute, College of Nursing and Health Sciences Flinders University Adelaide SA Australia; ^4^ Centre for Heart Rhythm Disorders University of Adelaide, and Department of Cardiology Royal Adelaide Hospital Adelaide SA Australia

**Keywords:** health behaviour, heart disease, hypertension, knowledge, nurse, risk factors

## Abstract

**Aim:**

The aim of this study was to explore awareness of heart disease and associated health behaviours.

**Design:**

A qualitative study was conducted using in‐depth interviews.

**Methods:**

The study participants were patients with hypertension. Data analysis was guided by Braun and Clarke's steps of thematic analysis and using NVivo12 software.

**Results:**

A total of 18 patients with hypertension were interviewed. The patients had poor understanding of heart disease and were not concerned about developing heart disease in the future. Barriers to fruit and vegetable consumption were poor access, cost and sociocultural factors, whereas being busy, poor physical health and lack of access to an exercise facility were barriers to physical activity.

## 1 INTRODUCTION

The burden of heart disease is increasing in developing countries against a background of limited awareness of associated risk behaviours (Ige et al., 2013). Despite this, its prevention, detection and treatment in developing countries are suboptimal (Cappuccio & Miller, 2016). Ethiopia is the second most populous country in Africa, with an estimated population of 110 million, and the country is in an epidemiological transition from a primarily infectious disease burden to long‐term disease burden, predominantly cardiovascular disease (CVD). The increasing burden of CVD is due to the ongoing improvements in socioeconomic status and life expectancy, increased urbanization and adoption of western lifestyles (Misganaw et al., 2014, 2017). Recent data show hypertension is a predominant factor for most patients presenting with cardiovascular disease who attend long‐term follow‐up care (Tefera et al., 2017). A review study revealed that the prevalence of hypertension in Ethiopia is 19.6% (Kelemu & Yonatan, 2015).

The burden of CVD in sub‐Saharan Africa is mainly associated with an increasing prevalence of multiple risk factors (Blokstra et al., 2012; Oluyombo et al., 2016). The prevalence of smoking in Ethiopia is 4.1%. Smoking cigarettes, *gaya* (a local water pipe used to smoke homemade tobacco products) and *hashish* (a drug made from the cannabis plant, typically inhaled in a pipe) are common practices in Ethiopia (Lakew & Haile, 2015). The World Health Organization (WHO) recommends that drinking no more than 10 standard alcoholic drinks per week and no more than 4 standard drinks on any 1 day reduces the risk of developing alcohol‐related harm. The rate of harmful alcohol consumption in Ethiopia is 8.94% (Ayano et al., 2019). A study revealed that 19% of known CVD patients are current alcohol drinkers (Negesa et al., 2019). In addition, khat chewing is a common unhealthy behaviour in eastern Africa; fresh leaves of the plant are chewed to achieve a state of euphoria and stimulation (Balint et al., 2009). However, it is associated with severe cardiac conditions such as acute myocardial infarction and cardiomyopathy (Al‐Motarreb et al., 2005; El‐Menyar et al., 2015). One in five known CVD patients is current khat chewers in Ethiopia. Moreover, more than half of CVD patients achieved a low level of physical activity (<600 MET minutes per week) (Negesa et al., 2019). Consumption of raw white meat is highly valued in Ethiopia; however, this increases the risk of heart disease due to its high cholesterol content (Seleshe et al., 2014).

The understanding of adults about CVD and its risk factors is low in developing countries (Akintunde et al., 2015; Aminde et al., 2017; Boateng et al., 2017; Monsuez et al., 2017; Tran et al., 2017). A review study showed the level of knowledge of CVD is suboptimal, as more than half of adults in sub‐Saharan Africa have poor knowledge of CVD. In addition, the level of knowledge of hypertension as a risk factor for heart disease is poor among adult populations in developing countries (Boateng et al., 2017). However, this review did not reveal patients' knowledge of particular risk behaviours, their risk perception and barriers to healthy behaviours. Studies have demonstrated that patients with hypertension who are at increased risk of developing heart disease do not perceive that they are at risk (Abed et al., 2015; Mazalin et al., 2015). A study from Seychelles identified one‐fifth of patients perceived they had a low cardiovascular disease risk irrespective of their actual risk (Alwan et al., 2009).

Overall, evidence demonstrating the prevalence of CVD and associated risk factors is increasing in sub‐Saharan Africa. However, none of these studies reported on patients' awareness of particular risk factors. There is a scarcity of data on patients' understanding of heart disease and associated risk behaviours in Ethiopia. The present study explored patients' understanding of heart disease and associated major risk factors. Thus, the findings of this study may be used to design interventional measures to improve awareness and reduce the burden of heart disease among patients with hypertension in developing countries particularly in Ethiopia.

## 2 AIMS

The aim of this study was to explore awareness of heart disease and associated health behaviours in patients with hypertension. In addition, the study also aimed to assess patients' self‐perceived heart disease risk and barriers to healthy behaviours, particularly physical activity and fruit and vegetable consumption.

## 3 METHODS

### 3.1 Design and settings

A qualitative study was conducted. The participants were patients with hypertension attending the follow‐up units of two main referral hospitals in eastern Ethiopia. These units provide treatment and secondary prevention services for patients with long‐term disease. Services include vital sign measurement, providing medications, assessing for complications and counselling about healthy lifestyles. The study was conducted from 5 May–27 June 2019.

### 3.2 Participants and recruitment

The study participants were patients with hypertension who were attending a long‐term follow‐up unit at one of the two hospitals during the study period. The participants were recruited in collaboration with the nurses or physicians who were on duty in the follow‐up departments. A purposive sampling technique was used to select study participants. Approximately 20 in‐depth interviews were planned based on Creswell's recommendation of 5–25 participants for qualitative study (Creswell, 1998). Data collection was continued until saturation of data was reached, and these principles were considered to determine the number of participants. Saturation of information was determined when the last three patients were unable to provide any new data to the researcher (Moser & Korstjens, 2018).

### 3.3 Inclusion criteria

Adult patients with hypertension who were in the age range of 18–64 years and attended a long‐term disease follow‐up department at one of the two hospitals were included in the study. Patients who had been on antihypertensive treatment for at least 2 months and had no confirmed diagnosis of heart disease were selected for recruitment in this study.

### 3.4 Data collection

A semi‐structured interview guide was developed based on the authors' previous study findings; thus, the themes were developed under the questions of interest (Negesa et al., 2019). The interview guide contained open‐ended questions, which were designed to explore patients' understanding of heart disease and its sign and symptoms, self‐perceived heart disease risk, perceived severity of heart disease, and understanding about heart disease risk factors. The English version of the interview guide was translated into local languages. The data were collected through face‐to‐face in‐depth interviews with patients who had a confirmed diagnosis of hypertension. The entire data collection was conducted by the principal author, who had previous experience of qualitative data collection and analysis. The interviews were conducted in the hospitals at a quiet place to avoid disturbance, to enable audio recording and to protect the privacy of patients. Each patient was given a code to keep the interview anonymous. The interviews took 30 min on average.

### 3.5 Ethical considerations

The study protocol received ethics approval from the Human Research Ethics Committee, University of Adelaide, and the Institutional Health Research Ethics Review Committee, Haramaya University. Written consent was obtained from each participant before starting the interview. The Health Belief Model (Glanz et al., 1997) was used to underpin the study.

### 3.6 Data analysis

The study is presented in line with consolidated criteria for reporting qualitative studies guidelines (Tong et al., 2007). In‐depth interview audio data were transcribed verbatim and then translated into English. Then, data files in Word documents were imported to QSR International's NVivo 12TM for thematic analysis. Data analysis was guided by Braun and Clarke's six steps of thematic analysis (Braun & Clarke, 2006). First, two researchers familiarized themselves with the data by reading and re‐reading through the translated data. Then, initial ideas were noted, codes were identified and similar codes were combined into sub‐themes and themes. A report was produced based on the final themes. Transcripts of the interviews were not returned to the patients as the study was anonymous, and thus, individual transcripts could not be identified.

### 3.7 Rigour

Lincoln and Guba's (1985) four criteria were used to establish the trustworthiness of the study. The researcher spent 3 months on data collection, and the research team included experts in critical care, qualitative research and a professor in cardiovascular nursing. The team had regular meetings to discuss and debrief on the progress of data collection. A study protocol with a detailed data collection procedure was developed, and data coding was checked for accuracy by the research team to maintain the dependability of the research. The research members checked the codes and themes that emerged, and the findings were reviewed by a panel who were familiar with the Ethiopian context. The use of purposive sampling and operationally defined data saturation (Moser & Korstjens, 2018) ensured the transferability of the study.

## 4 RESULTS

### 4.1 Participant characteristics

A total of 18 patients were interviewed. Their ages ranged from 31–64 years, and 11 were males. The majority of the patients had received follow‐up care for more than 2 years. About half started follow‐up care late in the progress of their disease. The sociodemographic characteristics of the interview participants are depicted in Table 1.

**TABLE 1:** Sociodemographic characteristics of patients

Variables (*N* = 18)	*N* (%)
Sex	
Male	11 (61.1)
Female	7 (38.9)
Age (mean ± *SD*)	55 ± 8
Education	
College or university completed	6 (33.3)
Secondary school completed	4 (22.2)
Primary school completed	3 (16.7)
No formal education	5 (27.8)
Occupation	
Government employee	5 (27.8)
Retired	5 (27.8)
Private	3 (16.7)
Housewife	2 (11.1)
Merchant	1 (5.5)
Nongovernment employee	1 (5.5)
Skilled private	1 (5.5)
Time since diagnosed with hypertension	
≥6 months	17 (94.4)
<6 months	1 (5.5)
Time since started follow‐up care	
≥6 months	16 (88.9)
<6 months	2 (11.1)

Questions were asked on following three main topics: (1) understanding of heart disease, (2) understanding of heart disease risk factors and (3) barriers to healthy behaviours. Themes were developed from the answers to these questions.

### 4.2 Patients' understanding of heart disease

#### 4.2.1 ‘*Lebe dikam*’, weakened heart

Patients with hypertension had a poor understanding of heart disease. But several described heart disease using its name in the Amharic language, ‘*Lebe dikam*’, which literally means ‘weakened heart’. Traditionally, this phrase was used to refer to any heart problem.

Since I don't have heart disease, there is nothing that I know about it. (P1)

It [heart disease] is ‘Lebe dikam’, it causes weakness, weakness of all body parts, it worsens health. (P12)

#### 4.2.2 ‘What has happened to my heart?’

Most patients recognized something was happening to their heart, with the most commonly recognized symptoms being fatigue, difficulty in breathing, increased heartbeat and sweating. But only a few of the patients identified chest pain, which is a common heart disease warning symptom. Several could not name a single sign or symptom of heart disease.

I feel my heartbeat is fast, but I don't know sign and symptoms of heart disease … I had no such problem previously. (P12)

However I don't have any understanding about heart disease, sometimes I feel my heartbeats very fast … sometimes I question myself … what happened to my heart? (P15)

#### 4.2.3 ‘I have not encountered it (hypertension) so far’

Even though all patients had been diagnosed with hypertension, most believed there is no relationship between this and heart disease.

I don't think hypertension causes heart disease, because I have not encountered it so far. (P17)

#### 4.2.4 ‘It is in the hand of God’

Most of the patients were not worried about developing heart disease. The patients' spiritual beliefs in the healings of ‘*tsebel*’, and a lack of understanding of hypertension as a heart disease risk affected their understanding of their risk of future heart disease. This was particularly evident in patients with no education.

I may not know what may happen in the future, it is in the hand of God … however, since I do exercise, I am not worried. (P14)

I don't have any heart problem so far, so I don't think about that [being worried about developing heart disease. (P1)

#### 4.2.5 ‘I am developing a heart problem’

However, several of the patients reported they were worried about their future heart disease risk. Some of them listed the symptoms they had experienced recently and were worried that they were developing or had already developed heart disease.

I drink coffee, chew khat and also I drink alcohol … I went to hospital for my blood pressure check‐up … doctor told me, I may develop another disease. I suspect that I am developing heart problem. (P11)

#### 4.2.6 ‘We live only as long as our heart is able to pump blood’

Interestingly, most of the patients perceived the consequence of heart disease to be severe illness and finally death. Most importantly, they believed that heart disease is worrying, hard to bear and difficult to live with. Moreover, they knew heart disease can occur suddenly, not allowing time to seek treatment and that delaying seeking treatment may cause sudden death.

Heart disease is very severe disease, because the heart is responsible for pumping blood for the whole body. For example, if a main water pump gets broken, the whole city will not get water at all. If a heart fails to pump, our body will not get blood at all, so we live only as long as our heart is able to pump blood. So, it is good to take care of the health of our heart. (P9)

### 4.3 Patients' understanding of heart disease risk factors

Overall, the patients had a reasonable understanding that smoking, alcohol consumption, inadequate fruit and vegetable consumption, and physical inactivity are risk behaviours for heart disease, but they had little knowledge about khat chewing and hypertension. Details on patients' understanding of individual risk factors are presented below.

#### 4.3.1 ‘Smoking affects the heart’

Interestingly, the patients consistently understood smoking substances containing nicotine, which results in addiction, could cause heart disease. Several of them expressed they had been smoking cigarettes for years. They also knew that shisha smoking causes heart disease in a shorter time than cigarette smoking. A few of them reported ‘*gaya*’ smoking, which is a traditional way of smoking tobacco in Ethiopia, is also associated with heart disease. Concurrent use of substances such as alcohol, khat, cigarettes and shisha is a common practice in Ethiopia.

I smoked cigarettes for 25 years, I quitted it now … When I was smoking, I had no understanding that cigarette smoking causes disease … I also didn't encounter any health problem at that time. (P15)

#### 4.3.2 ‘Alcohol causes heart disease’

Most of the patients understood excessive alcohol consumption is unhealthy. Most also understood locally prepared alcohol, ‘*areke*’, with 70% alcohol concentration is unhealthy.

Alcohol drinking can cause heart disease, it also causes hypertension. (P13)

#### 4.3.3 ‘Areke [local alcohol] is a medicine’

Several patients reported they were informed *areke* is a remedy for hypertension and they had it before breakfast to treat their high blood pressure. Despite their awareness, few patients self‐reported they still drink alcohol.

My friend told me ‘*areke*’ is a medicine, and I always drink two cups in the morning before breakfast. (P14)

#### 4.3.4 ‘I chew khat because everybody chews’

The patients' understanding of the relationship between khat chewing and heart disease was contradictory. Almost half of the patients reported khat chewing is not associated with heart disease. However, a few patients reported they were not sure about the relationship between heart disease and khat chewing, but they believed that those who chew khat have a tendency to smoke cigarettes and drink alcohol, which they knew are associated with heart disease. A few reported they still chew khat.

I am not sure about the relationship between heart disease and khat chewing … those who chew khat usually have a tendency to smoke cigarette and drink alcohol … there is a chain between them. They are interconnected, and in such a way, it may have an indirect effect. (P9)

#### 4.3.5 ‘A healthy diet’

All the patients understood that fruit and vegetables are healthy foods. However, they lacked understanding about the recommended daily amount of fruit and vegetables. Prominent vegetables mentioned by the patients were cabbages, potatoes and salad, whereas bananas, tomatoes and oranges were prominent fruits named by the patients. They also knew white meat, oil and butter‐rich foods are heart unhealthy. They identified that a high intake of salt and sugar is associated with heart disease. The patients stated ‘injera’ (a large sourdough flatbread), which is a common food in Ethiopia is healthy food.

Healthy diet is … diet with low cholesterol. White meat and butter oil are not advisable. Vegetable oil such as sunflower oil is good … fruit and vegetables are good for health of heart. ‘Injera’ is good for health, it contains iron and gives energy. Eating meat on a daily basis is dangerous for health of the heart, as it contains high cholesterol it affects the heart. So, a healthy diet is a diet which contains different types of food and less fat content. Salt should be very limited. (P9)

#### 4.3.6 ‘Strengthen body and heart’

Interestingly, most of the patients knew that exercise prevents heart disease, helps to control blood pressure and enhances effective heart function. They also believed lack of adequate exercise predisposed them to hypertension. Nevertheless, few of the patients could describe the relationship between exercise and heart disease.

Some people eat and sit the whole day. I have five brothers, they are farmers, and they are free of any health problem. I have a number of health problems; I have diabetes, hypertension, heart problem, nerve problem and a lot of things. Since I am retired from the military, I don't work, I sit the whole day and I just eat what I get. (P4)

### 4.4 Barriers to healthy behaviours

Given that inadequate consumption of fruit and vegetables and physical inactivity were the most prevalent risk behaviours among the patients, barriers to these behaviours are explored below.

#### 4.4.1 Barriers to fruit and vegetable consumption

##### ‘The problem is shortage’

Poor access to fruit and vegetables was one barrier to fruit and vegetable consumption. As Ethiopian agriculture depends on rainfall patterns, there can be shortages of fruit and vegetables particularly during the dry season.

I think the problem is shortage of fruit and vegetables in market. (P2)

##### ‘It is too expensive’

The increasing cost of fruit and vegetables and low income were barriers to fruit and vegetable consumption.

to buy fruit and vegetables, you need money, and nowadays it is costly to buy fruit and vegetables. For example, potato is 25 birr per kilo, cabbage, which we used to buy 5 cents, is more than 10 birr currently. My life situation does not allow me to eat fruit and vegetables as it is too expensive. (P7)

##### ‘Serve him meat and you are a good person’

Existing sociocultural beliefs are that eating meat especially white meat is considered a sign of good status and wealth. Fruit and vegetables are considered foods of the poor.

for example, if a guest come to your home, and you serve him fruit or vegetable, he will never consider he is served food, but if you serve him meat he will tell the whole village that you served him meat and you are good person. (P10)

#### 4.4.2 Barriers to physical activity

##### ‘I feel pain’

Physical health problems were one of the barriers to physical activity for several of the patients. The prominent physical problems identified as barriers were leg, back and chest pain.

If you go out early in the morning, 6:00 am, you will see a few individuals running on the road and in a stadium. However, I can't do running exercise, because I feel back pain. (P12)

##### ‘Because I feel tired’

Lack of time, being busy with routine activities and having a lot of commitments were identified as barriers to physical activity. The patients reported they were busy performing their daily activities and other extra commitments during the daytime and they felt too tired and exhausted to exercise after hours or in the morning.

Now I stop exercising, because I am a driver. Always I wake up early in the morning to go to work, I come back home at night‐time. I park my car 2 km away from my home. After parking my car, I catch a taxi to go home rather than walking, because I feel too tired to walk. (P15)

##### ‘Sometimes I am afraid’

Lack of accessible exercise facilities was one of the barriers to exercise. The patients reported there were either no exercise facilities or, if they existed, they were not adequate or too far away from where they lived. Most roads have no separate pedestrian walkway; thus, people share the road with vehicles. This results in fear of traffic accidents, which negatively affects patients' walking exercise behaviour.

There is no pedestrian walkway … sometimes I am afraid of a car accident. Where pedestrians walk is the same as the vehicles, and I catch a taxi rather than walking …. if there is a separate pedestrian walkway, I prefer to walk and take fresh air. (P15)

## 5 DISCUSSION

This study explored patients' understanding of heart disease and related health behaviours and perceived risks and barriers to health behaviours in a developing country, where there is a lack of literature. Thus, the findings have contributed to narrowing the evidence gap in this area and making recommendations for the implementation of evidence‐based health policy. The study highlights that patients' understanding of heart disease and associated risk factors is variable. Despite the presence of heart disease risk factors, patients had a deficient understanding of heart disease and failed to recognize their future risk.

Improving patients' health literacy is important to increase their understanding about heart disease and associated health behaviours (Aaby et al., 2017; Boateng et al., 2017; Mbambo et al., 2019; Tchicaya et al., 2018). The current study shows patients have inadequate knowledge about heart disease, despite being at high risk, and this is in accordance with findings from reviews of evidence from South Africa (Surka et al., 2015), Tanzania (Hertz et al., 2019) and sub‐Saharan African (Boateng et al., 2017). However, the current finding is inconsistent with a finding from the USA, which identified a high level of knowledge about heart disease among high‐risk patients (Tovar & Clark, 2015). This discrepancy could be due to differences in participant characteristics, particularly their educational attainment, as participants with high levels of education were recruited in the American study.

In addition, the patients in this study demonstrated a moderate knowledge of the main heart disease signs and symptoms, which concurs with findings from Canada which indicated adequate knowledge of heart disease warning signs among patients (Gill & Chow, 2010). Consistent with findings from Canada (McDonnell et al., 2014) and African‐based review findings (Boateng et al., 2017), only a few patients in this study identified chest pain as a main warning sign of heart disease.

For our participants, their concern about developing heart disease in the future is variable, and it was affected by sociodemographic factors. In particular, a low education level was related with low self‐perceived future risk. Despite the fact that hypertension puts patients at risk of heart disease, some patients in the current study underestimated their future risk, and this concurs with the findings of Price et al. (2009) from the UK and Frijling et al. (2004) from the Netherlands. Consistent with previous findings (Boateng et al., 2017; Peterson et al., 2012), the current research demonstrates that a higher education level is related to patients' better understanding of heart disease, its risk factors and self‐perceived future risk. A study from Spain (Perez‐Manchon et al., 2015) which demonstrated weak concordance between self‐perceived and actual cardiovascular risk among outpatients corroborates the findings of this study.

According to the Health Belief Model, patients' perceived severity and consequence of disease is one of the behaviour modifying factors (Glanz et al., 1997). Patients in the current study consistently understood the severity and the serious consequences of heart disease, and this aligns with findings from a Lebanese study where most patients perceived heart disease has serious consequence (Noureddine et al., 2013). A study conducted in Iran (Sabzmakan et al., 2014) where hypertensive patients considered cardiovascular disease to be very dangerous also confirms the finding of the current study.

Research has established that high blood pressure is a main driving factor of heart disease (Kokubo & Matsumoto, 2017). However, patients in this study had a variable understanding about the relationship between hypertension and heart disease, with some patients believing there is no relationship between heart disease and high blood pressure. Being aware of one's heart disease risk is an important factor in lifestyle change and self‐care behaviour (Glanz et al., 1997). However, difference in the methodology and participant profile makes it difficult to compare the current study with the findings of a review from sub‐Saharan Africa, which also demonstrated inadequate knowledge of hypertension as a CVD risk factor to confirm the findings of this study (Boateng et al., 2017).

Smoking is the most recognized heart‐unhealthy behaviour in the current study; almost all the patients identified smoking are heart unhealthy and predispose to heart disease. In support of this, it is also the least prevalent risk behaviour among known CVD patients. As we identified in our previous article, only 1% of CVD patients were current cigarette smokers (Negesa et al., 2019). A few patients in the current study reported they have ceased smoking as they understand it causes heart disease. The finding of Elshatarat and others that most CVD patients believe smoking causes heart disease supports this finding (Elshatarat et al., 2013). Consistent with this, numerous other studies identified smoking are commonly identified as a heart disease risk behaviour (Khan et al., 2006; Lechowicz et al., 2015; Surka et al., 2015).

Similarly, almost all the patients in the current study understood drinking alcohol causes heart disease, and this contrasts with previous findings (Whitman et al., 2015), which revealed most individuals perceive alcohol is heart healthy. The discrepancy could be due to differences in participant characteristics. Patients with hypertension used a traditional, locally prepared alcohol, *‘areke’,* which has a high ethanol concentration, to treat high blood pressure. This traditional alcohol is commonly considered a remedy for a number of diseases in Ethiopia, and thus, community education interventions are important to create awareness.

The participants in the current study varied in their understanding of the relationship between heart disease and khat chewing. Some patients know khat chewing causes heart disease whereas an equal number do not know. There is no similar study to make a comparison; however, review evidence shows khat chewing causes serious cardiovascular problems (El‐Menyar et al., 2015), and the increasing prevalence of khat chewing is a major public health challenge particularly in Ethiopia (Haile & Lakew, 2015). Existing cultural and religious beliefs in Ethiopia could have affected patients' understanding of the undesirable health effects of khat chewing.

Through our previous study, we identified that inadequate consumption of fruit and vegetables is the most prevalent risk behaviour among known CVD patients (Negesa et al., 2019). The current research investigated whether patients understood fruit and vegetables are healthy foods whereas fatty foods are unhealthy. The identified barriers to fruit and vegetable consumption were inadequate access, expense and sociocultural beliefs. Consistent with the findings of this study, previous studies demonstrated cost and lack of access are barriers to fruit and vegetable intake (Ashton et al., 2017; Hsiao et al., 2019). In addition, existing sociocultural beliefs that are passed down in families are major deterrents to fruit and vegetable consumption. In Ethiopia, eating meat has a sociocultural association with high status. Culturally, eating white meat has a special place for Ethiopians; thus, it is served on special occasions such as holiday events. Multi‐sectoral interventions are indeed important to improve sociocultural deterrents, access to and cost of fruit and vegetables.

Recognizing the poor exercise behaviour of the community and the rising prevalence of physical inactivity in Ethiopia is essential. Recently, government officials including the head of the Ministry of Health started a weekly mass sport programme in the capital, Addis Ababa, which has attracted and motivated many adults to exercise. As part of this programme, some parts of the roads will be free of vehicles and open for mass exercise. This sporting event should be expanded to cities across the country including Harar and Dire Dawa. Also, government officials promised to build sports fields in various districts to improve access. Having other commitments, poor physical health, lack of access to exercise facilities and easily accessible transportation were main barriers to physical activity for the patients. Consistent with the findings of the current study, Mbambo et al. identified that health problems and having no time are major barriers to physical activity in South Africa (Mbambo et al., 2019). In line with the findings of the current study, a study from Iran revealed tiredness, physical problems and being busy at work are barriers to physical activity among patients (Sabzmakan et al., 2014). To reduce the perceived constraints on physical activity, healthcare providers should take into account motivation and barriers to physical activity to tailor advice to patients' specific needs (Duclos et al., 2015). Thus, it is important to identify the barriers to physical activity to learn what to advise to overcome barriers to physical activity for individual patients.

Appropriate primary and secondary CVD prevention strategies should be implemented to reduce the burden of heart disease in Ethiopia. Interventions that improve health literacy should be designed and implemented to improve patients' understanding of heart disease and associated lifestyle behaviours. Implementation of effective and structured lifestyle education programmes is important to motivate patients to make lifestyle changes and to prevent serious complications of uncontrolled blood pressure such as heart attack and stroke. In addition, application of effective health behaviour change models can help to improve patients' perceptions and dietary styles (Horwath, 1999). Nurses have a responsibility to educate patients about the risk of heart disease from hypertension and preventive lifestyle behaviours. They should focus on intensive patient counselling and ensuring that patients understand the importance of adapting healthy lifestyle behaviour in heart disease prevention. Nurse‐led, intensive and structured counselling services may effectively improve awareness and promote healthy behaviour for patients. Thus, nurses should take a lead in interventions designed to improve patients' understanding and promote healthy lifestyle behaviour for patients.

Multi‐sector collaborative interventions are recommended to mitigate the multiple barriers to fruit and vegetable consumption and physical activity. Future research should estimate actual heart disease risk using an available future risk estimation such as Framingham Risk Score and associate it with self‐perceived risk and knowledge of heart disease risk factors. Interventional research and feasibility studies on innovative strategies are required to implement effective lifestyle interventions and to promote secondary prevention cardiovascular disease among patients in Ethiopia.

### 5.1 Limitations

This study was not free of limitations. The use of face‐to‐face interviews may increase social desirability bias, meaning the participants may have given a socially favourable response rather than their true thoughts or feelings. The results are specific for the investigated population and are not generalizable to the general population.

## 6 CONCLUSION

This study identified cultural barriers to heart‐healthy behaviours. Despite being at high risk of heart disease, the participants had a deficient understanding that hypertension predisposes to heart disease. Education level influences participants' understanding of heart disease and the associated risk factors. Healthcare workers, particularly nurses, need to identify and consider patients' understanding of health behaviours in planning secondary prevention strategies. Innovative education strategies are needed to improve patients' understanding of heart disease and risk behaviours and to overcome cultural barriers to health behaviours.

## CONFLICT OF INTEREST

The authors declare that they have no competing interests.

## AUTHOR CONTRIBUTIONS

LB: Design, data collection, analysis and writing of the manuscript. JM: Design, analysis and editing of the manuscript. PR: Design, analysis and editing of the manuscript. JH: Design, analysis and editing of the manuscript.

## Data Availability

The data that support the findings of this study are available from the corresponding author upon reasonable request.
